# 203. Screening and Targeted Prophylaxis for Clostridioides Difficile Infection: STOP-CDI

**DOI:** 10.1093/ofid/ofae631.061

**Published:** 2025-01-29

**Authors:** Matthew J Ziegler, Judith Anesi, Pam C Tolomeo, Laurel Glaser, Laura Cowden, Leigh Cressman, Elizabeth Huang, Alexa Patel, Ebbing Lautenbach, Brendan Kelly

**Affiliations:** University of Pennsylvania, Philadelphia, PA; FDA, Narberth, Pennsylvania; University of Pennsylvania, Philadelphia, PA; University of Pennsylvania, Philadelphia, PA; University of Pennsylvania, Philadelphia, PA; University of Pennsylvania Perelman School of Medicine, Philadelphia, Pennsylvania; University of Pennsylvania, Philadelphia, PA; University of Pennsylvania, Philadelphia, PA; University of Pennsylvania, Philadelphia, PA; Hospital of the University of Pennsylvania, Philadelphia, Pennsylvania

## Abstract

**Background:**

Patients receiving immunosuppression for oncology treatment and transplantation are at highest risk for developing hospital-onset *C.difficile* infection (HO-CDI). We studied a prophylactic enteral vancomycin intervention implemented to reduce HO-CDI during high-risk inpatient admissions among immunocompromised patients.

**Figure:**
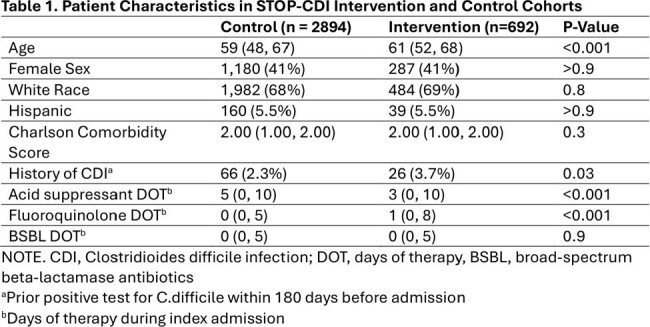

**Methods:**

The STOP-CDI intervention was implemented on 11/2021 at the Hospital of the University of Pennsylvania. Patients admitted for solid-organ transplant, autologous stem cell transplant, CART, or treatment for leukemia were screened for colonization with

*C.difficile*. Colonized patients were placed in contact isolation and were recommended oral vancomycin prophylaxis for a duration of 10 days or until discharge. Patients were compared to concurrent controls and historical controls in the two years prior to implementation of the STOP-CDI intervention. Concurrent controls were eligible for screening, but research staff were not available to perform screening. The primary study outcome was the development of HO-CDI, defined as a positive clinical diagnostic test after hospital day 3 but prior to discharge. Secondary outcomes included 90 day CDI, stool output, length of stay, VRE infection, and mortality. The impact of the STOP-CDI intervention was assessed using logistic and Poisson regression, with propensity-score weighting calculated using inpatient treatment category.

**Figure:**
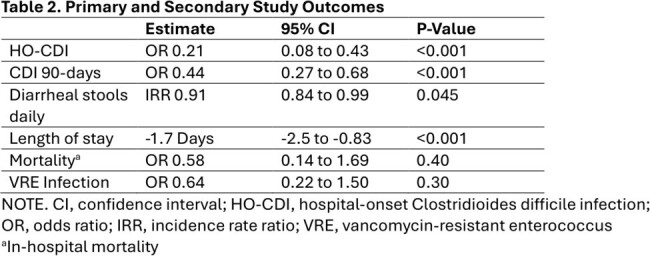

**Results:**

Between 11/2021 and 12/2023, 692 patients were screened for *C.difficile*, 11% were colonized. Characteristics of those enrolled and matched controls are displayed in Table 1. Compared to matched controls, the odds of HO-CDI was significantly lower in the intervention group (OR 0.21, P< 0.001) with a number needed to screen of 31 (Figure 1). We also identified significant reductions in 90-day HO-CDI, stool output, and length of stay (Table 2). There was no detected difference in VRE infection or mortality.

**Figure:**
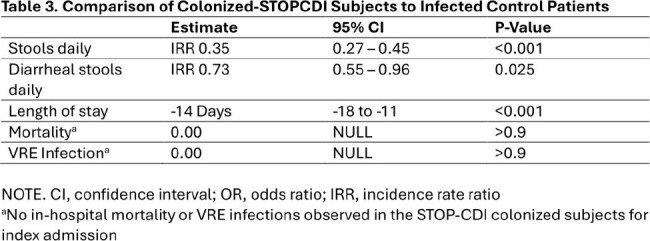

**Conclusion:**

The STOP-CDI intervention was effective at reducing HO-CDI, LOS, and symptoms of *C.difficile* in this high-risk cohort. Future work will assess the ability of a targeted screening and prophylaxis approach to reduce transmission of *C.difficile* on units and its utility in other patient populations.

**Figure 1.**
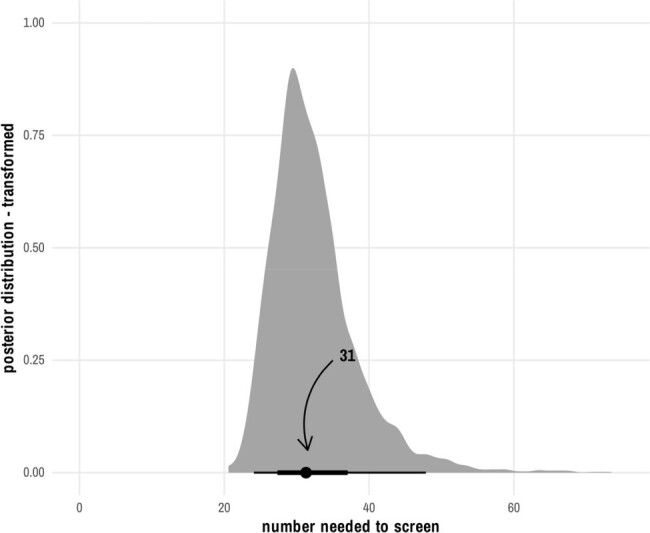
Number Needed to Screen to Prevent One Hospital-Onset C.difficile Infection.

**Disclosures:**

**All Authors**: No reported disclosures

